# Novel perspectives on the pharmacological treatment of thyroid-associated ophthalmopathy

**DOI:** 10.3389/fendo.2024.1469268

**Published:** 2025-01-13

**Authors:** Zilin Li

**Affiliations:** No. 1 Teaching Hospital, Norman Bethune College of Medicine, Jilin University, Changchun, Jilin, China

**Keywords:** thyroid-associated ophthalmopathy, drug treatment, research progress, molecular targets, traditional Chinese medicines

## Abstract

Thyroid-associated ophthalmopathy (TAO), an autoimmune disease closely related to thyroid dysfunction, remains a challenging ophthalmic condition among adults. Its clinical manifestations are complex and diverse, and disease progression can lead to exophthalmos, diplopia, exposure keratitis, corneal ulceration, and compressive optic neuropathy, resulting in irreversible vision damage or even blindness. Traditional treatment methods for TAO, including glucocorticoids, immunosuppressants, and radiation therapy, often have limitations and side effects, making this disease problematic in ophthalmology. As a result, the development of novel targeted drugs has become a research hotspot for addressing the pathogenesis of TAO. A range of novel targeted drugs, such as teprotumumab and tocilizumab, have been successfully developed and demonstrated remarkable efficacy in relieving inflammation and managing this disease. In addition, some drug candidates and molecular targets identified in the TAO *in vitro* model have shown promising prospects. This article briefly reviews the potential new strategies for future clinical treatment and the progress of new drug therapies for TAO.

## 1 Introduction

Thyroid-associated ophthalmopathy (TAO), also named Graves’ orbitopathy (GO) or Thyroid-eye disease (TED), is one of the most common orbital diseases in adults and occurs in approximately 20.1% of thyroid dysfunction patients (1). TAO is more frequent in women than men, approximately 4.9: 1 (2). This autoimmune disease is closely associated with thyroid disorders and involves various intraorbital tissues, such as the eyelid, extraocular muscle, and orbital adipose tissue. It is related to genetic factors, stress, radioactive iodine treatment, smoking, and other factors (3, 4). In the early stages of TAO, patients typically present with eyelid retraction, congestion, and edema of the eyelid and conjunctival tissues. Disease progression can lead to exophthalmos, ocular motility disorders, and diplopia. In later stages, exposed keratitis, corneal ulceration, and dysthyroid optic neuropathy (DON) result in irreversible vision loss or even blindness. Currently, we have some specifically designed targeted therapies for TAO, such as teprotumumab. However, these targeted drugs have not yet been widely used clinically. TAO remains a challenging problem in ophthalmology.

The pathogenesis of TAO remains incompletely understood. Current theories suggest that it is a complex organ-specific autoimmune disease arising from immune dysfunction triggered by antigenic stimulation, environmental factors, and genetics. These factors lead to an immune tolerance imbalance, activating autoimmune T cells (including Treg cells, CD8+ cytotoxic T cells, CD4+ T cells, natural killer T cells, Th1 cells, Th2 cells, and Th17 cells) and B cells (including Breg cells), which initiate a cascade of reactions (5–10). In patients with active inflammation, thyroid-stimulating hormone receptor (TSHR) expression in orbital tissues is significantly greater than in patients with quiescent inflammation (11). Additionally, insulin-like growth factor-1 receptor (IGF-1R) is overexpressed in the orbital fibroblast (OF) and lymphocyte of patients with Graves’ ophthalmopathy (12). TSHR and IGF-1R can form functional complexes that mediate downstream signaling via TSHR (13–15), suggesting that they may act as “co-culprits” in the pathogenesis of TAO (16).

**Figure 1:**
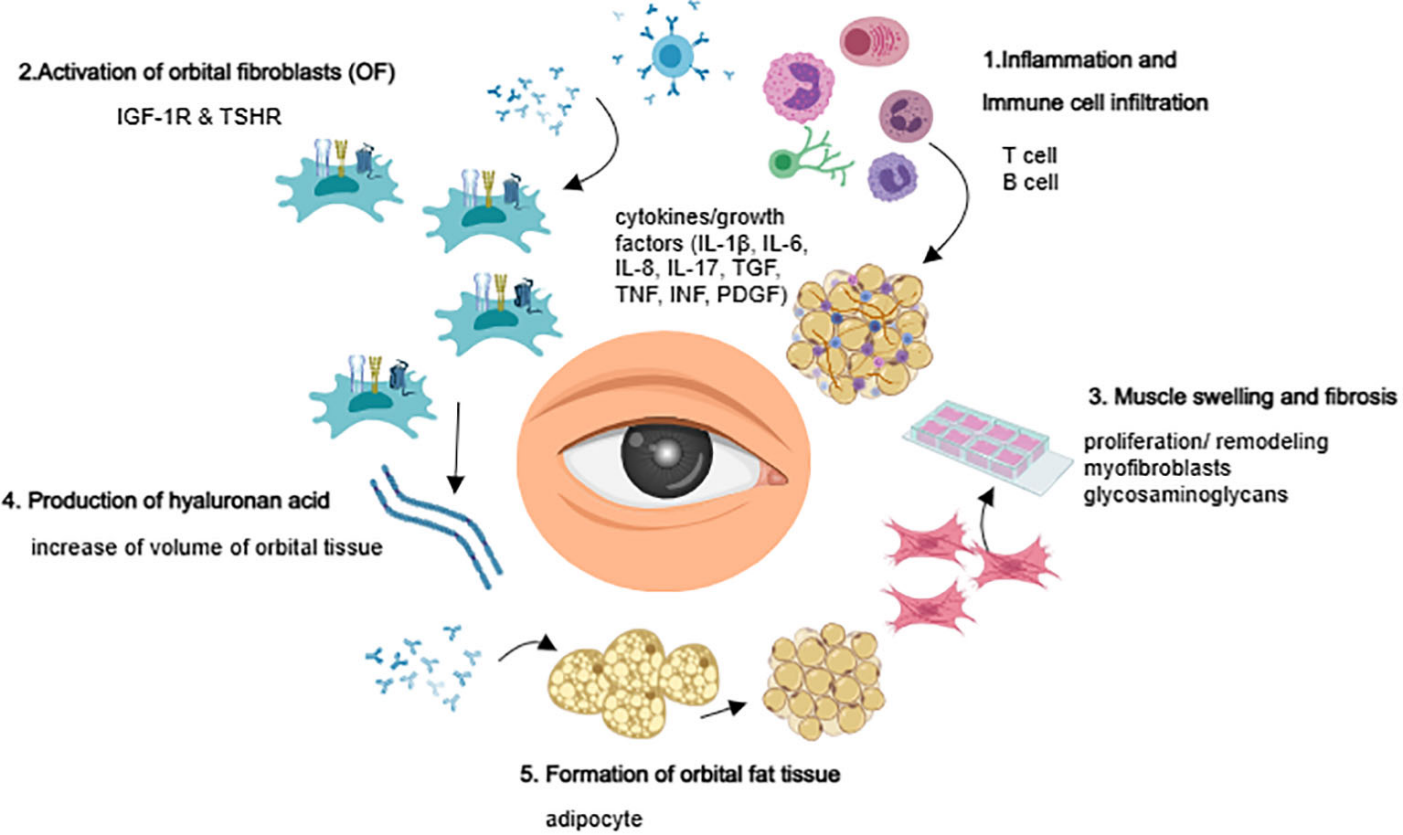
Overview of TAO pathogenesis.

OF plays a crucial role as both target and effector cells in the development of TAO (17, 18). OF interacts with infiltrating lymphocytes, monocytes, dendritic cells/macrophages, and mast cells in the orbit, and secrets various chemokines and cytokines, such as IL-1β, IL-2, IL-6, CXCL8, IL-10, and IL-17A (19); IL-35, IL-21, COX2, CCL2, CCL5, and TGF-β (20, 21); and IFN-γ and TNF-α (22). These cytokines amplify inflammation and autoimmune responses, stimulating OF to synthesize and secrete prostaglandin E2 (PGE2). PGE2 modifies B cell behavior, affects their phenotypic switch, and regulates mast cell activation. Furthermore, OFs secrete various glycosaminoglycans, particularly hyaluronic acid (HA), which forms high-molecular-weight polysaccharides with hydrophilic groups that promote orbital soft tissue edema. This leads to increased orbital pressure, exophthalmos, and swelling of the extraocular muscles. The increase in the number of orbital adipocytes, the expansion of soft tissues, and the secretion of adipokines and growth factors by adipose tissue may also contribute to the pathogenesis of TAO (23, 24) (**Figure 1**).

TAO treatment focuses on reducing orbital inflammation, alleviating exophthalmos, improving appearance, inhibiting the fibrosis of extraocular muscles, orbital fat, and periorbital soft tissues, and preserving visual function. Currently, the clinical management of TAO mainly involves nonspecific glucocorticoids (GCs) therapy, radiotherapy, specific targeted therapies (anti-IGF1R, anti-CD20) and surgical intervention. Current research hotspots include expanding the understanding of TAO pathogenesis, identifying relevant targets, and developing novel pharmacologic treatments. Herein, we comprehensively summarize the potential target strategies for the progress of new drug therapies and future treatment for TAO.

## 2 New drugs currently used in clinical practice

TAO currently includes clinical use of drugs such as immunosuppressants, somatostatin analogs, and monoclonal antibody-targeted biologics, most of which have shown gratifying benefits. Ongoing clinical trials evaluate these drugs’ efficacy and safety, which may soon become integral components of TAO management (**Table 1**).

**Table 1:** New drugs currently used in clinical practice.

Agents studied (no. of pts)	Mechanism of action	Dose and treatment protocol	Treatment indication	Effectiveness	Key findings of research
Mycophenolate mofetil (25)	antiproliferative effects on B and T cells	oral, 0.72 g daily	active moderate-to-severe TAO	Yes	The combination of mycophenolate mofetil and methylprednisolone as a first-line treatment.
Cyclosporin (26)	reduces T cell secretion of IL-2	oral, 7.5 mg per kilogram of body weight daily	moderate to severe TAO	Yes	The combination of cyclosporin and prednisone can be effective.
Azathioprine (27) Methotrexate (28)	interfering with the DNA/RNA synthesis of proliferating cells	oral, 100–200 mg daily; 12.5mg per week	active moderate-to-severe TAO	Yes	The combination with glucocorticoids represents a viable treatment option.
Lanreotide (29) Octreotide (30)	inhibit lymphocyte activation, proliferation, and cytokine production	im injection, 30mg every 2/4 weeks	mild active TAO	Conflicting	The clinical application of somatostatin analogs remains an area of ongoing exploration.
Teprotumumab (31)	IGF-1R monoclonal antibody	intravenous, 10 mg/kg for the first time, followed by 20 mg/kg	active TAO	Yes	The development of this drug represents a milestone in the treatment history of TAO.
Rituximab (32)	CD20 monoclonal antibody	intravenous, a single dose of 500 mg	active moderate-to-severe TAO	Conflicting	Rituximab has shown preliminary efficacy in TAO.
Tocilizumab (33)	IL-6 receptor monoclonal antibody	intravenous, 8 mg/kg body weight per month	moderate-to-severe corticosteroid-resistant TAO	Yes	Tocilizumab seems an effective and safe treatment option for refractory TAO.
Batoclimab (34)	a neonatal fragment crystallizable receptor (FcRn) inhibitor	subcutaneous injections, 680 mg,	moderate-to-severe TAO	Yes	The result highlights the efficacy and safety of batoclimab, endorsing its potential for further investigation for TAO.
Etanercept (35) Adalimumab (36) Infliximab (37)	tumor necrosis factor-alpha inhibitors	subcutaneous injection, 25 mg/80mg twice weekly	active, mildly-to-moderately severe TAO	Yes	These drugs may have a role in the treatment of active TAO with prominent inflammatory symptoms.
Statins (38)	activating TH2 and regulatory T cells,	oral, 20 mg daily	moderate-to-severe, active TAO	Yes	Addition of oral atorvastatin to an ivGCs regimen improved TAO outcomes.

TAO, Thyroid-associated ophthalmopathy; ivGC, intravenous glucocorticoids.

### 2.1 Immunosuppressant

Mycophenolate mofetil (MMF) competitively and reversibly inhibits inosine monophosphate dehydrogenase, reducing antibody production by B cells and having dual antiproliferative effects on B and T cells (39). Combined with GCs, MMF has been shown to enhance the treatment response rate among patients with active moderate-to-severe TAO (40, 41). A four-year retrospective cohort study confirmed that MMF is an effective and safe immunosuppressant for treating TAO (42). The clinical efficacy of active moderate-severe TAO patients was 100% (8/8) in non-DON group and 90% (9/10) in DON group at 24 weeks. In a clinical study involving 60 patients with active moderate-to-severe TAO, the efficacy of the MMF combined with GCs pulse therapy group (73.3% and 83.3%) at 12 weeks and 24 weeks, respectively, was significantly higher than that of the GCs pulse therapy alone group (46.7% and 60.0%) (p < 0.05) (43). In another comparative study involving 242 cases of TAO, the combined oral administration of MMF (one 500 mg tablet twice per day) and prednisolone (5 mg per day) was found to be highly beneficial in alleviating exophthalmos and diplopia (44). The latest EUGOGO guidelines also recommend the combination of MMF (0.72 g daily) and methylprednisolone as a first-line treatment for active moderate-to-severe TAO (25). Combined therapy is expected to provide greater benefits to patients.

Cyclosporin reduces T cell secretion of IL-2 by inhibiting the calcineurin pathway. Research suggests that combining it with GCs may be an effective method (56% improvement) for treating moderate to severe TAO, while cyclosporin monotherapy is not superior to GCs (22% *vs* 61%) (26, 45). Azathioprine is used alone to treat TAO, but its effectiveness is unclear. It may have potential benefits in reducing the recurrence of TAO after the cessation of steroids (27, 46). Methotrexate is an anti-folate antimetabolite that exerts immunosuppressive effects by interfering with the DNA/RNA synthesis of proliferating cells. It can improve periorbital and conjunctival edema, relieve exophthalmos, and reduce intraocular pressure in patients who are unresponsive to GCs (47, 48). Study showed that reduced GCs plus methotrexate therapy is effective and safer in treating active and moderate-to-severe TAO patients than 4.5 g GCs monotherapy (28). These investigations suggest that monotherapy utilizing immunosuppressants for the treatment of TAO has not demonstrated superior efficacy. When used in combination with GCs, they could provide good clinical benefits while reducing the side effects of GCs pulse therapy. Combination therapy represents a viable treatment option that merits consideration within clinical practice, albeit its long-term benefits necessitate further corroboration through extensive and rigorous clinical research endeavors.

### 2.2 Somatostatin analogs

Somatostatin is an endogenous cyclic peptide with widespread inhibitory effects on various systems (49). The rationale for using somatostatin analogs for treating TAO is based on their ability to inhibit lymphocyte activation, proliferation, and cytokine production (50, 51). Octreotide and lanreotide are considered first-generation synthetic somatostatin analogs. In a double-masked randomized controlled trial (RCT), octreotide did not demonstrate significant efficacy in patients (n=50) with moderate-to-severe TAO (p=0.043) (52). Chang TC et al. found that compared to placebo, lanreotide only alleviated diplopia when looking downward (p=0.03) and that lanreotide treatment did not significantly impact clinical activity scores (CAS) or proportion requiring orbital surgery (p=0.29) (29). In contrast, compared with placebo, octreotide has been shown to significantly reduce exophthalmos (p=0.027) at the end of the 4-month treatment period (30). Another double-blind RCT revealed that patients (n=25) treated with octreotide had more significant decreases in CAS (p= 0.02) and a significantly improved palpebral fissure height (decreased 1 mm on the right and 0.5 mm on the left, p<0.01) than the placebo group (53). The differences in conclusions between the studies may be attributed to variations in patient inclusion criteria, variations in oral dosage, as well as the small sample and short follow-up duration, leading to biases. Therefore, the clinical application of somatostatin analogs remains an area of ongoing exploration.

### 2.3 Teprotumumab

Research has indicated that IGF-1R is overexpressed in patients with TAO OFs and lymphocytes (12). Teprotumumab, a monoclonal antibody, binds to IGF-1R with high affinity and competitively inhibits its interaction with endogenous ligands (IGF-1 and IGF-2) (54, 55), demonstrating significant therapeutic potential. The development of this drug represents a milestone in the treatment history of TAO. Two multicenter, randomized, double-blind clinical trials (phase 2 and phase 3) (31, 56) evaluated the efficacy of intravenous teprotumumab compared to placebo (administered every 3 weeks for 8 times; the infusion dose was 10 mg/kg for the first time, followed by 20 mg/kg for subsequent times) in approximately 170 newly diagnosed active TAO patients. The results showed that compared with placebo, teprotumumab had better results with proptosis response (83% *vs* 10%), CAS of 0 or 1 (59% *vs* 21%), diplopia response (68% *vs* 29%), and TAO quality-of-life overall score (13.79 *vs* 4.43), and these results were maintained in most patients in the long term (p<0.001 for all). Severe adverse events were rare; the typical adverse events after teprotumumab treatment included muscle spasms (25%), nausea (17%), hair loss (13%), diarrhea (13%), fatigue (10%), hearing impairment (10%), and hyperglycemia (8%). Teprotumumab is contraindicated in patients with inflammatory bowel disease and pregnant women (57). The OPTIC-X study showed that TAO patients who did not respond to teprotumumab or whose disease worsened in the first course also benefited from teprotumumab treatment (58). More patients receiving teprotumumab achieved a reduction of at least 2 mm in proptosis at week 24 versus placebo (65 [77%] of 84 patients assigned teprotumumab *vs* 13 [15%] assigned placebo, p<0·0001) (59). Furthermore, compared to 20 patients in the placebo group, 42 patients in the group receiving teprotumumab exhibited significant improvement in ocular protrusion (-2.41 *vs* -1.48, p=0.0004) among long-term low-inflammation TAO patients regardless of disease duration/activity (60). Following treatment with teprotumumab, the lacrimal gland volume of TAO patients decreased significantly (768 mm^3^ to 486 mm^3^, p < 0.01), while dry eye symptoms decreased markedly (61). Although current dosing regimens have proven effective for treating TAO, further comprehensive research is warranted, including dose-ranging studies involving variable concentrations, infusion frequencies, and maintenance therapy durations.

### 2.4 Rituximab

Rituximab is a monoclonal antibody that targets CD20, which is only expressed by B cells (from pre-B cells to mature memory B cells). It is the first biological therapy applied to treat TAO (62). Mario Salvi et al. (63) reported using rituximab in 43 active GO patients, with 39 patients (91%) showing a tendency toward stability, 3 showing no change, and 1 showing disease progression. Subsequently, a double-blind RCT (64) compared the effects of rituximab and GC shock therapy in active moderate-to-severe patients, with the results showing that 100% of patients in the rituximab group experienced symptom alleviation at 24 weeks and that 38%-62% of patients experienced improvement in quality of life. However, another RCT conducted the same year (65) showed no additional benefit in active moderate-to-severe TAO patients using rituximab compared to those using placebo, accompanied by significant adverse reactions. In a study of rituximab treatment in 17 active moderate-to-severe GO patients, a low-dose (100 mg) single injection was effective (CAS score decreased from 4.56 ± 0.96 to 1.25 ± 1.14 at 24 weeks, p=0.001) (66). In a study of rituximab treatment for active moderate-to-severe TAO with different dosage groups (single dose of 100 mg, single dose of 500 mg, and 1000 mg given in two doses one week apart), considering factors such as patient activity remission, impact on quality of life, and treatment cost, it is recommended that most patients use a single dose of 500 mg (32). Rituximab has shown preliminary efficacy in the clinical treatment of TAO, but some studies report no significant benefits. This may be related to small sample sizes, varying inclusion and exclusion criteria, and differences in population types. Thus, further clarification of the role of rituximab in TAO treatment through long-term, large-sample, multicenter clinical trials is still needed.

### 2.5 Belimumab

Belimumab is a monoclonal antibody targeting B cell stimulating factor (BAFF). Like rituximab, belimumab targets naive and transitional B cells and is currently approved for treating systemic lupus erythematosus (67). Given the reported elevation of serum BAFF concentrations in Graves’ disease patients (68, 69) and the expression of BAFF on thyroid cells in patients with autoimmune diseases or a nodular goiter (70, 71), belimumab potentially exhibits beneficial effects in reducing disease activity in Graves’ disease and TAO. A clinical randomized controlled trial is underway in Italy (72), and the outcomes remain to be evaluated.

### 2.6 Tocilizumab

Tocilizumab is a recombinant human monoclonal antibody targeting the interleukin-6 (IL-6) receptor (73). IL-6 is a pleiotropic cytokine secreted by various cell types, including T cells, macrophages, fibroblasts, osteoblasts, and endothelial cells. A preliminary study showed that intravenous tocilizumab reduced disease activity in patients unresponsive to GC therapy (mean CAS score reduction 5.89 ± 1.41 points, p< 0.00027) (74). A subsequent double-blind RCT demonstrated a significant decrease in disease activity (CAS score < 3, 86.7% *vs* 35.2%, p =0.005) and severity in GC-resistant patients treated with tocilizumab compared to those treated with placebo (33). Two longitudinal studies (75, 76) also indicated that at least 4 months of tocilizumab treatment (one dose per month) provided significant benefits to patients with moderate-to-severe GC-resistant TAO. In an observational study of 12 patients with moderate-to-severe GC-resistant TAO, intravenous (tocilizumab 8 mg/kg, once every 28 days for 4 doses) reduced disease activity, with varying degrees of relief of proptosis and diplopia (Hertel score reduced by 2.3 mm on the right eye[p=0.003], 1.6 mm on the left eye [p=0.002]) (77). Tocilizumab appears to be an effective and safe treatment option for refractory TAO, providing a new alternative for patients with GC contraindications or resistance.

### 2.7 Batoclimab

Batoclimab, a neonatal fragment crystallizable receptor (FcRn) inhibitor, was evaluated in a double-masked RCT involving 77 patients with moderate-to-severe TAO (34). Subjects received weekly subcutaneous injections of batoclimab (680 mg, 340 mg, or 255 mg) or placebo. At 12 weeks, the batoclimab (680 mg) group showed a significant reduction in TSH-R-Ab levels, and participants tolerated the drug well throughout the study. Furthermore, phase 3 randomized placebo-controlled 24-week trials (NCT. 05524571 and NCT. 05517421) and a 24-week open-label extension (NCT. 05517447) have been designed, and more relevant clinical data are anticipated.

### 2.8 Tumor necrosis factor-related inhibitors

Key tumor necrosis factor α (TNF-α) inhibitors include etanercept (a recombinant human soluble TNF-α receptor fusion protein), adalimumab (a human monoclonal antibody targeting TNF-α), and infliximab (a monoclonal antibody against TNF-α). A preliminary study demonstrated that the subcutaneous etanercept injection improved ocular symptoms in 10 patients with active TAO without serious adverse events (CAS score reduced from 4 to 1.6 at 12 weeks, and the mean ophthalmopathy index reduced from 5.8 to 4.4) (35). Another case report described a female patient with concurrent TAO and rheumatoid arthritis who received etanercept for rheumatoid arthritis treatment, resulting in improved ocular symptoms and reduced exophthalmos (78). A retrospective study suggested that subcutaneous adalimumab might be an effective treatment for patients with active disease and significant inflammation (36), but prospective RCTs are needed to confirm its efficacy. Infliximab has also shown therapeutic effects in case reports of TAO, with a significant reduction in ocular inflammation, improvements in visual function, and no apparent short-term side effects (37, 79). However, large-scale clinical RCTs are still needed to assess the efficacy and safety of anti-TNF-α treatment.

### 2.9 Statins

Statins are commonly used as lipid-regulating agents in clinical practice. Previous studies have suggested that statin therapy may be associated with a reduced risk of TAO development in Graves’ disease patients (80). A Swedish study (81) statistically analyzed 34,894 patients newly diagnosed with Graves’ disease and revealed that statin users had a significantly lower likelihood of developing TAO, indicating a potential preventive effect on TAO progression. There is a significant correlation between the occurrence of TAO and total cholesterol and low-density lipoprotein cholesterol levels, suggesting that statin treatment may benefit patients with TAO and provide a new therapeutic direction (82). A phase 2 clinical trial in Italy showed that adding oral atorvastatin to intravenous GC pulse therapy improved TAO outcomes in patients with moderate-to-severe active ophthalmopathy and hypercholesterolemia (p=0·042) (38). This may be due to the ability of stains to shift the primary proinflammatory T-cell response to an anti-inflammatory response by activating TH2 and regulatory T cells, thereby alleviating clinical symptoms. Future phase 3 studies are required to confirm this association.

## 3 Promising candidates target drugs in the laboratory

With continuous exploration by researchers, it has been confirmed that some commonly used clinical drugs and traditional Chinese medicine components have therapeutic effects, such as inhibiting adipogenesis, anti-inflammatory, and anti-fibrotic effects, in the TAO *in vitro* model. The following section will briefly discuss the new perspectives of old drugs, as well as emerging molecular targets in TAO.

### 3.1 Drug discovery in an *in vitro* model of TAO (I)

Preventing orbital tissue inflammation and adipose tissue hyperplasia during the early stages of the disease is critical for addressing exophthalmos. Previous studies have reported that several clinically used drugs exhibit therapeutic potential for inhibiting adipogenesis and inflammation in *in vitro* models of TAO. Diclofenac suppresses COX-2 to inhibit mature adipocyte formation in OFs (83). Salazosulfamide inhibits adipogenesis by downregulating PPARγ expression in OFs (84). Idelalisib, a treatment for lymphocytic leukemia, inhibits lipid droplet formation and decreases the expression of PPARγ and c/EBPα/β in OFs (85). Simvastatin inhibits the expression of adipogenesis-related genes in preadipocytes and OFs stimulated with cigarette smoke extract (86). Metformin, a first-line treatment for type 2 diabetes, inhibits adipogenesis and inflammation in *in vitro* models of TAO by activating AMPK phosphorylation and suppressing ERK phosphorylation signaling (87). Nintedanib, a treatment for idiopathic pulmonary fibrosis, inhibits fibroblast growth factor-induced adipogenesis in OFs from patients with TAO (88). Disulfiram, a drug used to treat alcohol aversion, significantly inhibits the differentiation of OFs from TAO patients into mature adipocytes, inhibiting ERK phosphorylation by partially inhibiting ALDH1A1 (89). Linsitinib, a novel and highly selective dual inhibitor of IGF-1R and insulin receptors, may reduce local inflammatory responses by promoting Treg differentiation and/or activation and reducing TNF-α levels in a mouse model of TAO (90). Intriguingly, caffeine has also been found to reduce oxidative stress and inhibit adipogenesis in an *in vitro* model of TAO (91).

Studies have shown elevated levels of platelet-derived growth factor (PDGF)-AA, AB, and BB in the orbital connective tissue of TAO patients, with OFs expressing PDGF receptors (92, 93). PDGFs can induce the proliferation of OFs, promote adipogenesis (94), promote the secretion of hyaluronic acid and IL-6 (95), and increase the expression level of surface TSHR (96). Tyrosine kinase inhibitors, such as imatinib, dasatinib, and nilotinib, may be candidates for targeting PDGF signaling (97). The serum IL-17 concentration and positive detection rate are significantly greater in TAO patients than in healthy controls, and the serum IL-17 concentration is significantly associated with CAS (p < 0.001) (98). The proportion of T cells that secrete IL-17A is significantly greater in TAO patients than in healthy subjects (19, 99). Various IL-17A monoclonal antibodies, including secukinumab (100), ixekizumab (101), and bimekizumab (102), have been successfully developed. They are expected to serve as targets in future treatment approaches.

Traditional Chinese medicines (TCMs) have also been found to inhibit adipogenesis. Quercetin is used as an adjunctive treatment for chronic bronchitis and coronary heart disease, and it inhibits the differentiation of OFs into mature adipocytes by reducing oxidative stress (103). Tanshinone II inhibits adipogenesis in OF in a dose-dependent manner, reducing oil red O staining and decreasing the expression of PPARγ and C/EBPα (104). Icariin, a flavonoid, affects the differentiation of TAO preadipocytes into mature adipocytes by inhibiting AMPK/mTOR-mediated autophagy, reducing orbital adipose tissue expansion and lipid droplet accumulation (105). Chloroquine/hydroxychloroquine, an antimalarial drug, affects the proliferation, adipogenesis, and hyaluronic acid production of TAO OFs by inhibiting autophagy (106). Liensinine, an alkaloid extracted from lotus seed embryos, inhibits IL-13-induced autophagosome formation and the overexpression of autophagy markers, increases the LC3-II/LC3-I ratio, and ultimately downregulates p62 expression in TAO OFs, thereby reducing inflammation and adipogenesis (107). Berberine, a significant component of Coptis chinensis, inhibits lipid droplet formation in OFs and reduces IL-1β-induced inflammation (108).

### 3.2 Drug discovery in an *in vitro* model of TAO (II)

Suppressing the fibrosis of extraocular muscles and periorbital soft tissues is another urgent clinical issue. Pirfenidone, a treatment for idiopathic pulmonary fibrosis, inhibits IL-1β-induced increases in TIMP-1 and hydroxyproline levels in an *in vitro* model of TAO, exerting antifibrotic effects (109). Pirfenidone has also been found to regulate TGF-β1-mediated OF differentiation into myofibroblasts and ECM homeostasis (110). Simvastatin has been found to inhibit TGF-β-mediated α-SMA expression in an *in vitro* model of TAO (111). Metformin exerts anti-inflammatory and antifibrotic effects on OFs by activating autophagy and the AMPK/mTOR signaling pathway (112). Relaxin, a recombinant form of the human hormone relaxin-2, alleviates TGF-β1-induced α-SMA, COL1A1, FN1, and TIMP1 expression in OFs through the Notch pathway, exerting antifibrotic effects (113).

Certain TCMs have also been found to have antifibrotic effects. Curcumin inhibits the TGF-β1 signaling pathway and attenuates TGF-β1-induced CTGF and α-SMA expression (114). Quercetin has been shown to inhibit TGF-β-stimulated FN1 and collagen Iα expression and suppresses MMP2 and MMP9 activity in an *in vitro* model of TAO, suggesting potential therapeutic and preventive uses for chronic fibrosis (115). Dihydroartemisinin may exert antifibrotic effects on OFs through the MAPK/ERK and STAT3 signaling pathways (116). Lutein reduces TGF-β1-induced α-SMA and COL1A1 expression by inhibiting the ERK/AP-1 pathway (117). Crocin suppresses TGF-β1-induced OF proliferation and migration, exerting antifibrotic effects by inhibiting the ERK and STAT3 signaling pathways (118).

These encouraging findings broaden our clinical therapeutic approaches. The safety of commonly used Western medicine and TCMs active ingredients has been widely validated in clinical practice. However, most of these studies involved *in vitro* experiments; therefore, whether these drugs can also inhibit adipogenesis and reduce tissue inflammation and fibrosis in TAO animal models remains unverified. Furthermore, there is a lack of an in-depth understanding of drug interactions with systemic conditions (thyroid function indicators, liver and kidney function) and potential systemic adverse effects during TAO treatment. Therefore, there is still a long way to go before the clinical application of TAO therapy. Next, we will outline several new drug treatments and their recent advancements in clinical settings.

### 3.3 Advances in other molecular targets

With further research, the discovery of emerging molecular targets has revolutionized therapeutic strategies for TAO. Precisely targeted molecules are expected to reduce therapeutic side effects, enhance treatment efficacy, and offer new hope for patients. Human placenta-derived mesenchymal stem cells were shown to inhibit lipid accumulation and ameliorate adipogenesis in the orbital tissues of TAO model mice (119). Fingolimod (a sphingosine-1-phosphate inhibitor) improves the outcome of experimental Graves’ disease and TAO by modulating the autoimmune response to TSHR (120). IL-38 has been shown to exert anti-inflammatory and antifibrotic effects in *in vitro* models of TAO (121). IL-11 promotes OF fibrosis by increasing the phosphorylation of ERK and STAT3 and upregulating the expression of the fibrosis-related proteins α-SMA and COL1A1 (122). TGF-β has been shown to enhance YAP expression, and YAP silencing or inhibition with cerivastatin, verteporfin, TED-347, and CA3 has been shown to significantly reduce myofibroblast differentiation and collagen formation in TGF-β-induced TAO (123). The expression of BRD4, a member of the bromodomain and extra terminal family, is upregulated in the orbital tissues of TAO patients and in TGF-β1-stimulated TAO OFs. BDR4 may regulate the fibrotic process in the OFs of TAO patients through the FoxM1/Plk1 axis, and targeting the BD2 domain of BRD4 may exert antifibrotic effects (124). TMEM2 has been shown to suppress inflammatory cytokine production, ROS production, lipid droplet formation, and adipogenic factor expression in OFs in TAO mouse models (125). GSDMD-mediated OF cell pyroptosis induces TAO inflammation through the NF-κB/AIM2/caspase-1 pathway (126).

**Figure 2:**
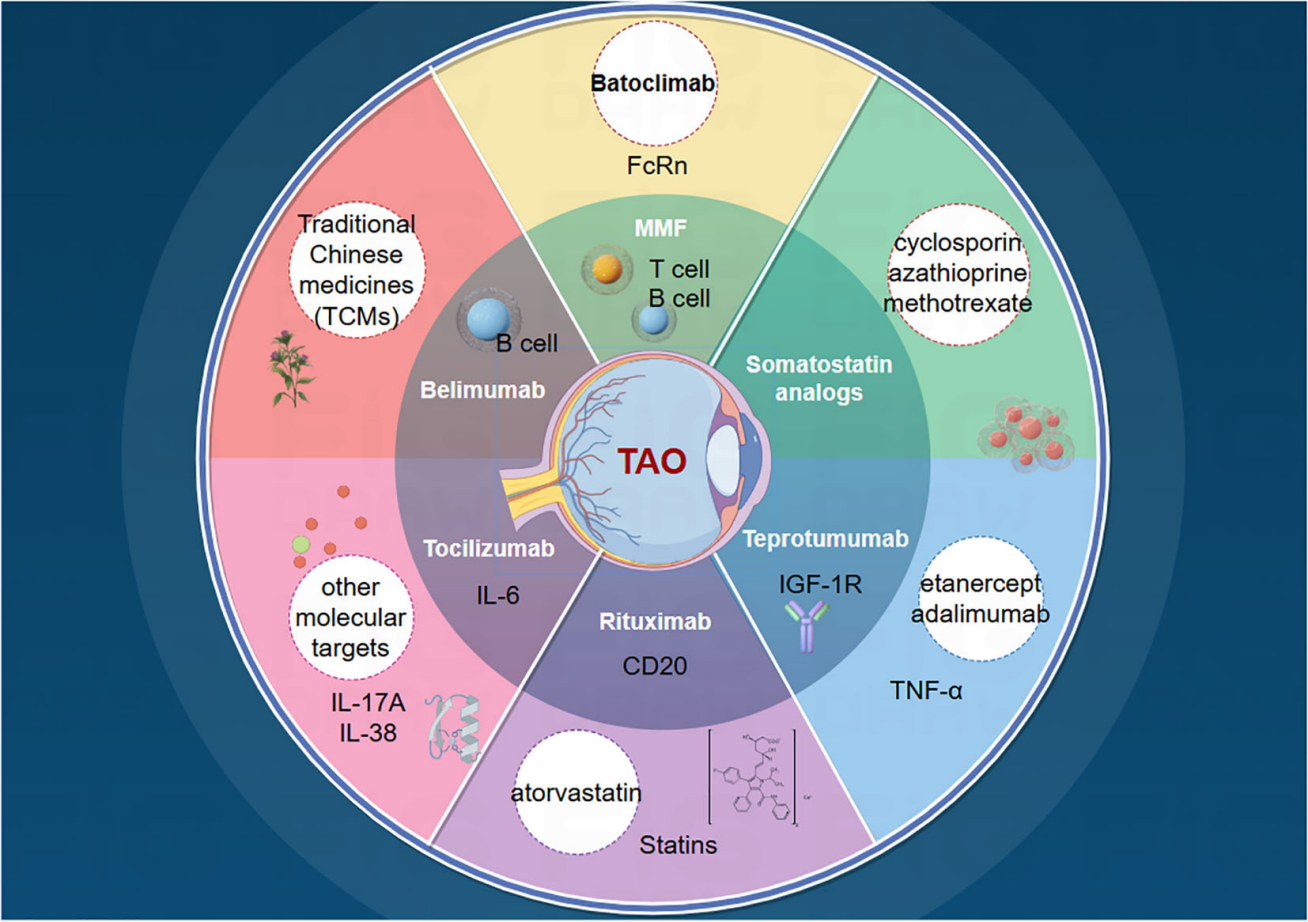
Current status of TAO drug therapy and future directions.

Numerous challenges remain. First, the functions and mechanisms of these targets and small-molecule compounds may not be fully elucidated in laboratory settings, adding uncertainty to subsequent drug development. Second, experimental targets may lack sufficient clinical data, making it difficult to predict their efficacy and safety in humans. Additionally, potential issues such as stability, specificity, and interactions with other biomolecules require further investigation and validation of new targets. Therefore, despite promising new targets for TAO treatment, additional research and overcoming these limitations are necessary. With technological advancements, we anticipate the discovery of more targets and the elucidation of their mechanisms of action, leading to more precise and effective TAO treatment options (**Figure 2**).

## 4 Discussion

As research on TAO has progressed, the limitations of traditional treatments have spurred the emergence of novel therapeutic approaches, with various new drugs gradually entering clinical trials. Currently, some medications can alleviate the activity and severity of the disease, but there are still few prospective, multicenter, randomized controlled clinical studies. Further research is needed to investigate the effectiveness, safety, optimal dosage, and administration methods of these drugs. The clinical application of these drugs should be guided by specific indications personalized according to the disease grade and activity of each patient. However, the exact mechanisms of these drugs and the risk of complications during treatment remain unclear, thus limiting their clinical use.

Given the complexity and individual differences in the pathogenesis of TAO, personalized treatment plans are crucial. MMF combined with GCs pulse therapy and intravenous teprotumumab can be considered effective methods for current drug therapy. Early intervention can significantly impact the disease course, improve long-term prognosis, and reduce the incidence of severe complications. It is imperative to understand the pathogenesis of TAO further, identify relevant targets, and develop new treatment strategies. Multitarget combinations, new administration methods, and traditional Chinese medicine components may offer promising avenues for improving drug efficacy and reducing risks. However, high-quality RCTs and the elucidation of the exact underlying mechanisms are still needed. There is an urgent need to develop eye drops, ophthalmic gels, and sustained-release formulations for periorbital injection incorporating contemporary polymer-based medical materials.

In conclusion, drug therapy for TAO is evolving from new perspectives. We eagerly await the clinical application of more effective, safer, longer-lasting, and more straightforward drugs or treatment modalities, aiming to maximize the long-term prognosis and quality of life of patients with TAO.
